# Transverse myelitis in myelin oligodendrocyte glycoprotein antibody-associated disease

**DOI:** 10.3389/fneur.2023.1210972

**Published:** 2023-07-06

**Authors:** Gina Perez-Giraldo, Natalia Gonzalez Caldito, Elena Grebenciucova

**Affiliations:** Department of Neurology, Feinberg School of Medicine, Northwestern University, Chicago, IL, United States

**Keywords:** transverse myelitis, MOG myelitis, myelin oligodendrocyte glycoprotein associated disease, MOG antibody positive myelitis, MOGAD myelitis

## Abstract

Transverse myelitis (TM) is the second most common presentation of myelin oligodendrocyte antibody-associated disease (MOGAD), occurring in approximately 26% of affected patients. The diagnosis may be complicated by the lack of diagnostic specificity of low titers of MOG antibody in serum, fluctuation in seropositivity overtime, including initially normal MRI in up to 10% of patients, and in many instances complete resolution of radiological abnormalities when MRI is done in a significantly delayed fashion. The use of preventive disease modifying treatments is limited by the uncertainty whether the disease process will remain monophasic or become relapsing, as well as by the lack FDA approved treatments. In this review, we discuss clinical, radiological and cerebrospinal fluid (CSF) characteristics, including the significance of MOG titers and changes in the seropositivity status for the diagnosis of MOGAD-associated TM, its radiological features and management options, highlighting the data on the risk of relapses associated with TM at presentation and the need for further randomized clinical trials to empower effective treatment algorithms.

## Introduction

Transverse myelitis (TM) is the second most common presentation of myelin oligodendrocyte antibody-associated disease (MOGAD), occurring in approximately 26% of MOGAD (1). TM can occur in isolation, simultaneously with optic neuritis (less than 10% of patients) or as part of acute disseminated encephalomyelitis (ADEM) (2–4). Clinical presentation in children and adults may vary. TM in MOGAD can occur shortly after an infectious illness (1). It is therefore not surprising that rare cases of MOGAD TM have been reported both after SARS-CoV2 vaccination (ChAdOx1 nCoV-19) and after the infection itself (5, 6). The severity of the attack varies, typically moderate to severe with EDSS scores >4 in about half of patients (7) with up to one third being nonambulatory at the nadir of acute myelitis attack (8), and many requiring bladder catheterization (9). In a prospective UK cohort, transverse myelitis as part of MOGAD was found to be less common in children as compared to adults, and relapsing TM was only observed in adults (3). Optic neuritis is the most common relapsing phenotype of MOGAD, and patients with TM at onset have less risk of relapse as compared to those with other MOGAD phenotypes (3).

## Clinical features of MOGAD-associated TM

The clinical manifestations of MOGAD myelitis include sensory, motor or bowel-bladder symptoms, erectile dysfunction, typically with acute to subacute onset of paraparesis or quadriplegia (10). Motor and sensory symptoms are usually bilateral, and sphincter dysfunction is more common than in aquaporin 4 antibody (AQP 4 antibody) positive NMOSD (4). Patients may also present with acute flaccid myelitis (AFM) and may be initially thought to have post-viral or viral-induced AFM (8, 11). Tonic spasms and severe neuropathic pain are less common with MOGAD as compared to AQP4 positive NMOSD (12). Prognosis is generally good, with excellent motor recovery, but residual neurogenic bladder and sexual dysfunction can occur (2).

### Diagnosis of MOGAD myelitis

MOGAD myelitis is diagnosed when a patient has neurological deficits and tempo of symptomatic development compatible with myelitis, a clear positive serum MOG-IgG test, and supportive of diagnosis MRI features (2). The latest international expert consensus advocates towards the use of live cell-based assays to increase diagnostic specificity. If not available, fixed cell-based assays can be used, with a clear positive result being a titer >1:100 (2). Titers lower than 1:100 have a lower predictive value (number of true-positive results/total positive results) for MOGAD and may lead to misdiagnosis. For example, a titer of MOG of 1:20 to 1:40 carry a positive predictive value of 51%, meaning that nearly 50% of patients with this titer may have a different etiology of their clinical presentation (13). Serological testing should ideally occur before administration of corticosteroids, intravenous immunoglobulins, or plasma exchange, as these interventions can increase the risk of false negative result. In cases of a high clinical suspicion but a negative test, if done after initiation of immune therapies, testing should be repeated about 3 months or later. Screening for the presence of serum MOG-IgG routinely in patients with clear features of multiple sclerosis is not recommended, as false positives can occur, decreasing the positive predictive value of the test and leading to misdiagnosis (2, 10).

In addition, titers of MOG antibody can fluctuate with intermittent seroconversion to negativity and can become undetectable over time (14); this, and the fact that the MRI abnormalities can disappear (up to 72% of the brain lesions; 79% of spinal cord lesions) (13, 15, 16), make a retrospective diagnosis of MOGAD impossible in some scenarios, which warrants a continuity of high degree of suspicion if new neurological symptoms arise (17).

Cerebrospinal fluid (CSF) analysis typically demonstrates lymphocytic pleocytosis, with >50 WBC/mm^3^ seen in about 30% of patients (9). Oligoclonal bands are frequently absent, with positivity rates being less than 10%–15% (4, 18). MOG-IgG antibody concentrations in CSF are typically low, however in some cases MOG-IgG can be positive in CSF and not in serum. Intrathecal antibody production occurs more frequently in MOGAD than in AQP4-positive NMOSD (19). Caution is advised in interpretation of the MOG-IgG positivity when clinical features are atypical of MOGAD, as false positives can occur. Testing both CSF and serum is not recommended for routine evaluations (2). However, if the serum MOG antibody is negative in a patient presenting with clinical, radiological and CSF findings typical of MOGAD, CSF testing for MOG antibody may be considered, as well as re-testing serum several months later.

Current diagnostic criteria for MOGAD require exclusion of an alternative diagnosis. Differential diagnostic considerations include multiple sclerosis, neuromyelitis optica spectrum disorder, CRMP-5 antibody positive paraneoplastic myelopathy, GFAP antibody positive encephalomyelitis, neurosarcoidosis, SLE associated TM, ischemic myelopathy, metabolic myelopathy, infections and neoplastic causes. Specific findings pointing to other inflammatory diagnoses are summarized in Table 1.

**Table 1:** Differential diagnosis of MOGAD associated-TM.

Disorder	Multiple sclerosis	MOGAD	Aquaporin 4 positive NMOSD	Neurosarcoidosis	NeuroBehcet’s	Anti-GFAP astrocytopathy	Anti-CRMP-5
Onset	Acute/subacute	Acute/subacute	Acute	Subacute/chronic	Acute/subacute	Acute/ subacute	Subacute
Evolution	Relapsing/progressive	Monophasic/relapsing	Relapsing	Progressive	Progressive	Progressive	Progressive
Clinical cues	Sensory symptoms predominantly.	Bladder/bowel symptoms.	Area postrema syndrome Other autoimmune comorbidities. (Ex SLE, Sjogren’s)	Evidence of systemic involvement. (Ex: Lymphadenopathy)	History of recurrent oral or genital ulcers.	Associated with AQP4 ab and NMDA ab. Concomitant ovarian teratoma.	History or increased risk for cancer. Constitutional symptoms.
Laboratory							
Biomarkers	n/a	Anti-MOG ab in serum > > CSF	Aquaporin-4 ab in serum	n/a	N/A	Anti-GFAP ab in CSF > serum	Anti-CRPM5 ab in serum and CSF
CSF cells	Pleocytosis <50cells/μL (mainly lymphocytes)	Pleocytosis (mainly lymphocytes, neutrophils present in over 40%)	Pleocytosis (neutrophils predominant early on)	Pleocytosis (mainly lymphocytes)	Pleocytosis (neutrophils predominant)	Pleocytosis >50cells/μL (lymphocytes, monocytes)	Pleocytosis
O-bands unique to CSF	>80%	<10%	<20%	<20%	Rare	50%	Can be present
Neuroimaging							
Extension	Short/long if confluent lesions	LETM, short lesions may be present, conus medullaris involvement	LETM, up to 15% with short segment lesions	Short/LETM	Short/LETM	LETM	LETM
Number of lesions	Single/multifocal	Single or multifocal	Single	Single/multifocal	Single/multifocal	Single	Single
Location	Dorsal/lateral column	Central (30% only gray matter) H sign	Central (gray and white matter)	Central / subpial/ leptomeningeal involvement	Central or anterior horn cells	Diffuse	Tract specific (lateral columns)
Enhancing	Nearly always in the acute setting	Enhancing in 50% cases acutely	Yes (most commonly in optic neuritis and transverse myelitis) Variable incidence of brain enhancing lesions	Yes, usually persistent for months.	Yes	Often faint enhancement	Variable
Enhancing pattern	Ringlike or homogeneous	Faint; dorsal nerve root enhancement, cauda equina and pial enhancement may occur	Ringlike or patchy “cloud-like” Pencil-thin linear enhancement of the ependymal surface of lateral ventricles	Dorsal subpial (trident sign)	Ringlike or non-specific (bagel sign)	Patchy, punctate and pia	Tract specific

NA, not applicable; MOG, Myelin oligodendrocyte glycoprotein; MOGAD, Myelin oligodendrocyte glycoprotein antibody disease (MOGAD); ab, antibody; NMOSD, Neuromyelitis optica spectrum disorder; SLE, systemic lupus erythematosus; CSF, cerebrospinal fluid; GFAP, Glial fibrillary acidic protein; anti-CRPM-5, collapsin response-mediator protein-5; NMDA, N-methyl-D-aspartate; LETM, Longitudinal extensive transverse myelitis; O-bands, oligoclonal bands.

### Radiological features of MOGAD myelitis

**Figure 1:**
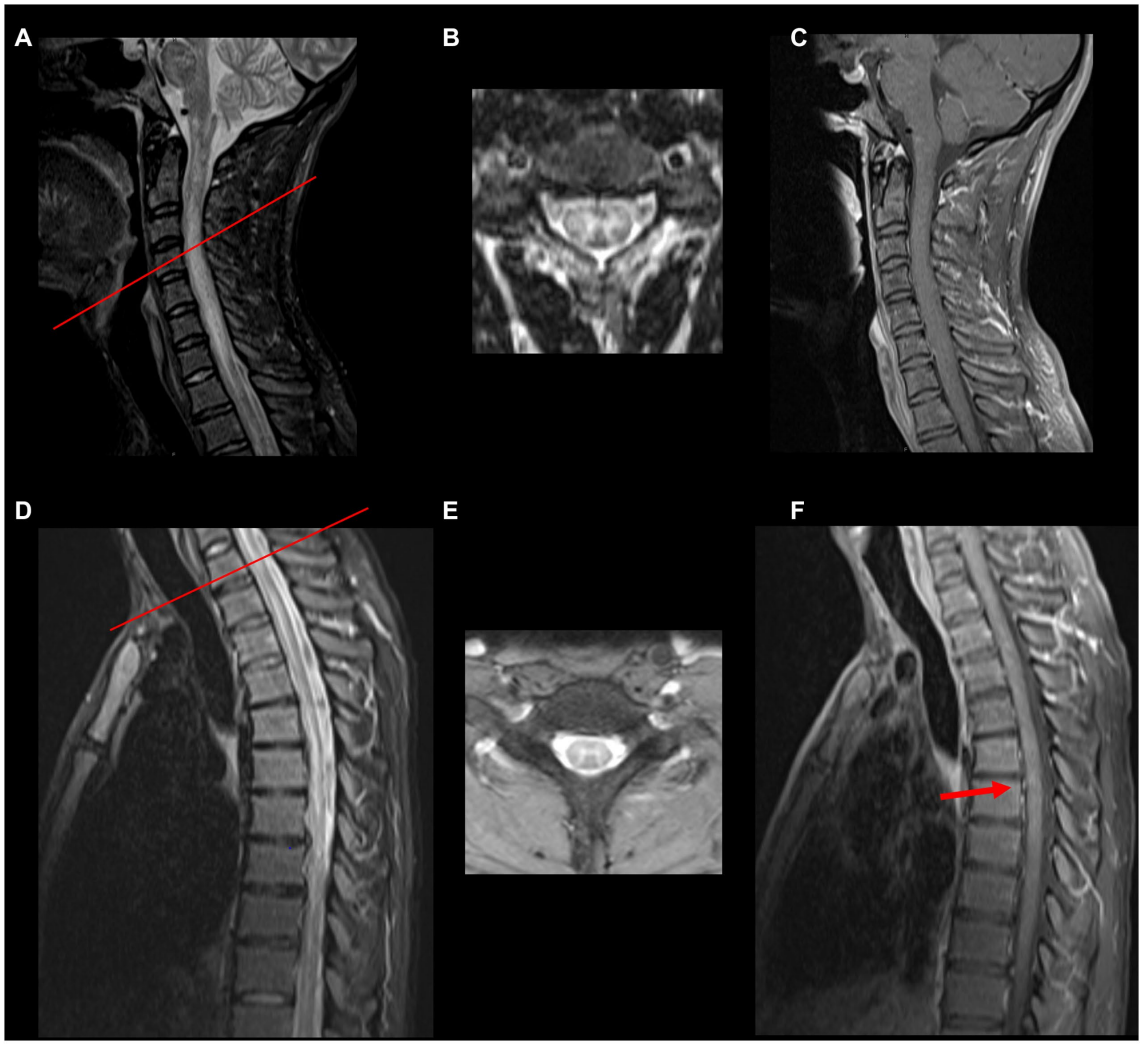
Spinal cord MRI in MOGAD myelitis. **(A,D)** Abnormal hyperintense T2/FLAIR signal abnormality with cord expansion from the cervicomedullary junction extending to the thoracic spinal cord. **(B,E)** Show axial T2 images (red bar indicates the spinal cord of the axial section) resembling H sign (grey matter involvement). **(C,F)** Show post gadolinium T1 sequences, respectively. No enhancement is noted in the cervical spinal cord, but a small focus of enhancement is noted at T9 and T10 (red arrow).

Acute myelitis due to MOGAD is typically longitudinally extensive involving 3 or more vertebral segments in length (Figure 1). Short lesions can occur in about 7% of patients, more commonly in those patients that are approximately 40 years of age (3). Multifocal spinal cord lesions can occur (2, 18). Spinal cord necrosis is typically not seen (20). Some T2 signal abnormalities may be initially subtle and contrast the severity of clinical presentation. In fact, in some cases linear T2 signal abnormalities may be difficult to distinguish from a prominent central canal. In these instances, carefully evaluating axial images may be helpful, as well as repeating the MRI at a later time to evaluate for the evolution of the lesion. MRI of the brain may also show MOGAD-associated lesions and should be considered in TM evaluation. Some patients with MOGAD TM may also have clinically silent spinal cord lesions (20). Although current data are limited by the retrospective nature, one study found silent spinal cord MRI lesions occurred in none of the patients during remission and in up to 7.3% of those during acute attack (21). Another study confirmed that finding new or enlarging spinal cord lesions inter-attack is rare and occurs in <1% of patients (22). Initial MRI of the spinal cord may be normal in up to 10% of patients, and repeat MRI within days would be warranted (13–15).

A negative MRI in a patient presenting acutely with clinical features of myelitis should not deter from testing for MOG antibody. The spinal cord lesions are typically located in the central cord, can involve both the gray and white matter, affect more than 50% of the axial section or can be restricted to the grey matter in about 50% of patients, which on axial imaging can be seen as the “H sign” (2, 23). T2 signal abnormality confined to gray matter (sagittal line and axial H sign) as well as lack of enhancement favor diagnosis of MOGAD over NMOSD or MS (8).

A straight T2 hyperintense line with surrounding hyperintense signal in the anterior and posterior gray matter horns on sagittal view can also be seen in MOGAD myelitis and is known as pseudo dilation of the central canal (23). The conus medullaris can be affected in about 26% of patients with MOGAD (24), which is a valuable radiographic clue, as the conus medullaris is less frequently affected in other demyelinating conditions, such as multiple sclerosis (MS) and neuromyelitis optica spectrum disorder (NMOSD), with rates of involvement as low as 1.3 and 6%, respectively, (8, 24–26). Gadolinium enhancement can occur in about half of patients with MOGAD myelitis and can be heterogeneous giving a cloud-like appearance (2, 9, 23). Interestingly, dorsal nerve root enhancement, cauda equina and pial enhancement can also occur (2).

Clinically silent new lesions on MRI are rare and occur in only 3% of patients with MOGAD (21, 27). Upon follow up and resolution of acute myelitis, complete resolution of spinal cord lesions can be seen in up to 79% of MOGAD cases (4). Spinal cord atrophy can be seen in severe cases, but this is rare (2).

### Acute management of MOGAD transverse myelitis

In the acute setting, patients presenting with TM receive intravenous corticosteroids. For those with incomplete recovery or severe clinical picture overall, plasma exchange is typically used next (9, 28). Intravenous immunoglobulins (IVIG) can also be used following the plasma exchange completion, given that intravenous immunoglobulins can also serve as an effective preventive therapy for those patients who elect long-term preventive therapy option (29). Steroids are typically tapered slowly over at least a month or longer, with some data pointing to a lower risk of relapse in those with steroid use >1 month (16).

### Chronic management of MOGAD

#### Disease modifying treatments

In about 40%–50% of cases, MOGAD appears to be monophasic long-term; however, 50%–60% patients presenting with their first attack of MOGAD will go on to develop the relapsing type of the disease. While the disappearance of MOG antibody serologically cannot be used as a definitive sign of the disease being monophasic (17), some data show that pediatric patients who seroconvert to negativity may have a somewhat lower risk of relapse (30). In the UK cohort, the final status of MOG antibody was not found to be associated with the relapsing disease overall, but the longitudinal analysis showed a reduction of 4–5% in monthly relapse risk in those who seroconverted negative for MOG IgG. Same study showed that the patients presenting with TM may have lower rates of relapsing disease: TM as the first attack was associated with a lower risk of relapse (OR, 0.03; 95% CI, 0.004–0.23; *p* = 0.001) and a longer time to first relapse (HR, 0.42; 95% CI, 0.22–0.82; *p* = 0.011) (16). In another study, TM at presentation alone or in combination with another syndrome (ON, ADEM, brainstem) was likewise associated with lower risk of relapse (HR, 0.41; 95% CI, 0.20–0.88; *p* = 0.01) (3).

A discussion whether a long-term disease modifying treatment needs to be started after the first attack or only after the disease course proves itself to be relapsing, risks versus benefits of both approaches should be carefully explored with every patient. Some clinicians may offer preventive disease modifying treatments as early as after the first attack, if the attack was severe with poor recovery leading to significant residual disability, thus if the second attack were to occur, it would be detrimental to the patient’s quality of life and independence.

Treatments commonly used in the prevention of MOGAD relapses are rituximab, azathioprine, mycophenolate mofetil, tocilizumab, and intravenous immunoglobulins. A recent meta-analysis of 41 studies (3 prospective, 1 ambispective, 37 retrospective) evaluating efficacy of MOGAD-associated treatments found that the proportions of patients free of relapse were 65% [95% confidence interval (CI): 49%–82%] on azathioprine, 73% (95% CI: 62%–84%) on mycophenolate mofetil, 66% (95% CI: 55%–77%) on rituximab, 79% (95% CI: 66%–91%) on IVIG, and 93% (95% CI: 54%–100%) on tocilizumab (31).

#### Symptomatic management

Patients with MOGAD transverse myelitis can go on to develop chronic neuropathic pain, weakness, and bladder dysfunction. Addressing these symptoms effectively and in a timely manner is critical to the patient’s quality of life. Medications utilized for the treatment of neuropathic pain such as gabapentin, pregabalin, and in some instances addition of duloxetine can be utilized. Physical and occupational therapy during recovery period and periodically in those without complete recovery are recommended. Bladder symptoms are best addressed and managed by a knowledgeable urologist or a neuro-urologist. Neuropsychiatric consultation may be warranted in patients with adjustment disorder, anxiety or depressive symptoms secondary to the medical condition.

### MOGAD TM outcomes

Neurological outcomes of patients with TM due to MOGAD are typically more favorable than of those with anti-aquaporin4 antibody positive NMOSD. A recent study evaluating long-term outcomes of patients with TM due to MOGAD (*n* = 32) vs. NMOSD (*n* = 57) found that MOGAD TM patients on average had a lower EDSS score than patients with AQP4-Ab TM (1.8 [1.0–8.0] vs. 3.0 [1.0–8.0]), reflecting better outcomes. Due to higher predilection of MOG positive TM to conus localization, persistent bladder dysfunction was more common in patients with MOGAD (59% with MOGAD and 48% with AQP-4 positive NMOSD), with up to 23% requiring long-term catheterization in both groups. In addition, neuropathic pain was less common in patients with MOGAD TM vs. NMOSD TM (29% vs. 13%) (32).

## Discussion

Transverse myelitis is the second most common presentation of MOGAD, with a substantial number of patients presenting with para- or quadriplegia at nadir of the attack. Although generally purporting better outcomes than AQP 4 antibody positive NMOSD, some patients are left with persistent bladder dysfunction, erectile problems, and weakness. The diagnosis is often complicated by the lack of diagnostic specificity of low titers of MOG antibody in the serum, its serologic fluctuation from positive to negative and back to positive, disappearance in some cases when diagnostic workup is delayed by months, including initially normal MRI in up to 10% patients, and resolution of radiological abnormalities when MRI is done in a significantly delayed fashion. Moreover, many patients with low titer of MOG antibody in serum such as 1:20 or 1:40 are initially misdiagnosed with MOGAD and may go one to develop other conditions such as MS or NMOSD, among others. Long-term management of MOGAD-associated TM is complicated by the uncertainty as to whether the disease process will remain monophasic or become relapsing. Because MOG antibody titers can fluctuate and go from positive to negative to positive again, basing the risk of relapse purely on a decrease in titer or seroconversion to negativity does not appear to guarantee monophasic course of the disease. Recent data suggest that male gender and TM at onset may overall have a lower risk of relapse; however, small patient numbers, and the length of follow up continue to be important limiting factors. Because at least 40%–50% cases will remain monophasic, most clinicians will start preventive disease modifying treatment only if the disease proves to be of relapsing phenotype; others may choose to start disease modifying treatments after the first attack in those with poor response to initial treatment and significant residual disability, in fear that the second attack might be devastating to what may already be a significant neurological disability with poor quality of life and reduced independence. Currently there are no FDA approved drugs specifically targeting MOGAD. Despite several off label management options discussed above, some patients may relapse through multiple disease modifying therapies. Randomized controlled clinical trials are crucially needed to create an evidence-based treatment algorithm for those affected by MOGAD.

## Author contributions

GP-G and EG made a substantial, direct, and intellectual contribution to the work. NC provided MRI imaging Figure 1 and Table 1 with legends. All authors approved the submitted version.

## Conflict of interest

EG has served on advisory boards and received honoraria from Horizon Therapeutics, Alexion, Genentech, Prevail Therapeutics and has received research support from NIH and Genentech.

The remaining authors declare that the research was conducted in the absence of any commercial or financial relationships that could be construed as a potential conflict of interest.

## Publisher’s note

All claims expressed in this article are solely those of the authors and do not necessarily represent those of their affiliated organizations, or those of the publisher, the editors and the reviewers. Any product that may be evaluated in this article, or claim that may be made by its manufacturer, is not guaranteed or endorsed by the publisher.
